# Antibodies to *in silico* selected GPI-anchored *Theileria parva* proteins neutralize sporozoite infection *in vitro*

**DOI:** 10.1016/j.vetimm.2018.03.004

**Published:** 2018-05

**Authors:** James Nyagwange, Vishvanath Nene, Stephen Mwalimu, Sonal Henson, Lucilla Steinaa, Benjamin Nzau, Edwin Tijhaar, Roger Pelle

**Affiliations:** a International Livestock Research Institute (ILRI), P. O. Box 30709, Nairobi, Kenya; b Cell Biology and Immunology Group, Wageningen University, The Netherlands; c Biosciences Eastern and Central Africa – International Livestock Research Institute (BecA-ILRI) Hub, P. O. Box 30709, Nairobi, Kenya

**Keywords:** *Theileria*, Sporozoites, Antigens, Neutralizing antibodies, Vaccine

## Abstract

East Coast fever (ECF) caused by *Theileria parva* kills cattle in East, Central and Southern Africa leading to significant economic losses. Vaccination is used as a control strategy against ECF and is presently dependent on deliberate infection with live sporozoites and simultaneous treatment with a long-acting oxytetracycline. Although effective, this method has serious limitations; the immunity is parasite strain specific and immunized cattle can become life-long asymptomatic carriers of the parasite, posing risk for the spread of the disease. In efforts to develop a subunit vaccine, the role of antibodies in the neutralization of *T. parva* sporozoites infection of host cells has been investigated and a circumsporozoite protein, p67, is able to induce such neutralizing antibodies. However, the p67 protein only protects a proportion of immunized cattle against *T. parva* challenge and such protection might be improved by inclusion of additional parasite antigens that neutralize sporozoite infection. In an attempt to identify such antigens, we searched the re-annotated *T. parva* genome for genes predicted to contain GPI anchor signals, since they are likely to be located on the cell surface, and expressed fragments of six of the selected genes in *E. coli*. The recombinant proteins were used to raise antisera in mice. Antisera to two proteins, TpMuguga_01g00876 and TpMuguga_01g00939, neutralized sporozoite infectivity to a high degree, while antisera to two additional proteins, TpMuguga_01g00095 and TpMuguga_04g00437, exhibited moderate neutralizing capacity. We conclude that these four antigens are potential vaccine candidates, which should be evaluated further in cattle.

## Highlights

• Six *in silico* selected GPI anchored proteins expressed in *E. coli*.
• Murine antisera against four of expressed proteins neutralizes sporozoite infection.
• Four novel antigens that are promising vaccine candidates against East coast fever identified.

## 1 Introduction

East Coast fever (ECF) caused by *Theileria parva* is prevalent in East, Central and Southern Africa where it causes significant losses by reducing cattle productivity and kills a large number of them (Nene et al., 2016). The disease is of major economic importance because of the high mortality it causes, and the expensive measures used to control the tick vector. In the 1900s, Dr. Arnold Theiler identified the three-host life cycle tick, *Rhipicephalus appendiculatus,* as the chief vector for transmission of *T. parva,* which occurs trans-stadially (Norval et al., 1992). The sporozoites, which are the mammalian infective stage of the parasite develop in the tick salivary glands and are introduced into the bovine host during tick feeding (Shaw, 1996). The sporozoites enter the host lymphocytes rapidly by a zippering process of the host and sporozoite cell membranes (Fawcett et al., 1982b; Shaw, 1996). Once inside the lymphocytes, the sporozoites differentiate into schizonts that undergo several multiplication cycles (Shaw, 2003). A proportion of the schizonts undergo merogony resulting in the production of merozoites that invade erythrocytes and develop into piroplasms. These piroplasms are the tick infective stage and after uptake during blood feeding they will restart the life cycle of the parasite (Shaw, 2003). Blocking sporozoite proteins involved in the lymphocyte invasion process, such as p67, presents a vaccine control strategy for ECF. The p67 protein, named for its size ∼67 kDa protein, is the major surface antigen of sporozoites and the primary target of monoclonal antibodies that neutralize sporozoite infectivity in *in vitro* assays (Dobbelaere et al., 1984; Musoke et al., 1984; Dobbelaere et al., 1985).

Apart from controlling the tick vectors by acaricides, infected cattle can be treated and burpavaquone has remained the commercial drug of choice three decades after its discovery (McHardy et al., 1985). However, the drug needs to be administered early in infection in order to be effective (Babo Martins et al., 2010) and resistance has been reported in *Theileria annulata* (Mhadhbi et al., 2010), which raises concerns for future ECF control as resistance could occur in *T. parva*. A live vaccine, based on an infection and treatment method (ITM) is also used to control ECF (Radley et al., 1975a; Radley et al., 1975b). It involves infection with live sporozoites and simultaneous treatment with a long-acting oxytetracycline (Radley et al., 1975b). The drug controls but does not kill the parasite allowing generation of protective acquired immunity (reviewed in Nene et al., 2016). However, the generated immunity is strain specific and animals vaccinated using the ITM can become life-long carries of the parasite, posing risk for spread of the disease (Uilenberg, 1999). Production of the vaccine from infected ticks is also very laborious and the vaccine requires a liquid nitrogen cold chain for delivery making it expensive (Uilenberg, 1999).

The protection conferred by the ITM vaccination is mediated by major histocompatibility complex (MHC) class I-restricted cytotoxic T lymphocytes (CTL) (Morrison and Goddeeris, 1990; Morrison, 2009). The sporozoites injected in the animal differentiate into schizonts and produces a transient parasitosis resulting in induction of specific MHC class I-restricted CTL that are directed against the schizont infected lymphoblasts (Morrison and Goddeeris, 1990). These cellular responses were established in experiments that passively transferred immunity from immune animals to their naïve twins by transferring thoracic duct leukocytes from the former to the latter (Emery, 1981). It was later determined that immunity was related to CD8^+^ cells as demonstrated by transfer of efferent lymph CD8^+^ cells enriched by monoclonal antibody mediated complement lysis of CD4^+^ cells, γd T-cells and B-cells (McKeever et al., 1994). However, there is indirect evidence for a role of antibodies in mediating immunity to ECF derived from observations that animals that survive repetitive challenge with infected ticks either in the field or experimentally develop sporozoite neutralizing antibodies (Musoke et al., 1982). Monoclonal antibodies against p67, a circumsporozoite protein, also neutralizes sporozoite infection *in vitro* (Dobbelaere et al., 1984; Musoke et al., 1984) and experimental vaccines based on this protein has shown partial protection (Musoke et al., 1992; Hall et al., 2000; Bishop et al., 2003). The p67 based vaccine might be improved by including additional sporozoite antigens.

In order to identify vaccine candidate antigens that might neutralize sporozoite infectivity, we performed a bioinformatics search of the re-annotated *T. parva* genome (cited in Tretina et al., 2016) for proteins predicted to contain a C-terminal Glycosylphosphatidylinositol (GPI) anchor signal and/or N-terminal signal peptide. GPI-anchored proteins are usually expressed on the cell surface where they are involved in extracellular interaction (Ferguson, 1999). Proteins with signal peptides are usually destined to the secretory pathway (von Heijne, 1990). Therefore, proteins with these features are likely to be located on the cell surface and are likely vaccine candidates to induce sporozoite neutralizing antibodies. Structurally, the proteins are linked via the C-terminal to ethanolamine with a phosphodiester bond linking the core glycan (tri-mannoside glucosamine), which in turn is linked to inositol phospholipid (Ikezawa, 2002). GPI-anchored proteins are ubiquitous among eukaryotic species and play different roles including infection (Tachado et al., 1996; Delorenzi et al., 2002) and can elicit strong immune responses, making them targets of vaccine development (Gilson et al., 2006). We report on the expression of six of the *in silico* selected GPI anchored proteins and neutralization of sporozoite infection by antisera raised against four of the recombinant proteins.

## 2 Materials and methods

### 2.1 *In silico* analysis and selection of genes encoding GPI-anchored protein

We performed a bioinformatics search of the re-annotated *T. parva* genome (cited in Tretina et al., 2016) for proteins predicted to contain a C-terminal GPI anchor signal and/or an N-terminal signal peptide using PredGPI (Pierleoni et al., 2008) and SignalP 4.1 (Petersen et al., 2011), respectively. Following PredGPI analysis, GPI-proteins were sorted based on their Hidden Markov Model (HMM) scores in decreasing order of prediction accuracy, from highly probable, probable and weakly probable. The selected proteins were further analyzed for the presence of predicted N-terminal signal peptide.

To determine if the selected genes are conserved across various isolates of *T. parva,* DNA sequence reads for the genomes of 16 *T. parva* isolates for which data is available in the European Nucleotide Archive (ENA) were mapped to the re-annotated *T. parva* Muguga reference genome (cited in Tretina et al., 2016) using the smalt short read aligner (www.sanger.ac.uk/resources/software/smalt) set at default settings. Duplicates were marked using Picard Tools (http://broadinstitute.github.io/picard) set at default parameters. FreeBayes (Garrison and Marth, 2012) was used for calling single nucleotide polymorphisms (parameters: −K −i −X −u −q 20 −min-coverage 6). SNPs were annotated using snpEff (Cingolani et al., 2012).

### 2.2 Sporozoite RNA preparation and cloning of gene fragments

The procedure for sporozoites production has been described before (Patel et al., 2016) and we have recently reported on DE-52 column purification of *T. parva* sporozoites (Nyagwange et al., 2018). RNA was extracted from the sporozoites using high pure RNA isolation kit (cat no. 11828665001; Roche) and primers used in RT-PCR reaction (one step RT of 3 mins at 95 °C, 30 cycles of 30 s at 95 °C, 60 s at 60 °C, 60 s at 72 °C and final elongation of 5 mins at 72 °C). We designed primers (Supplementary Table S1b) to amplify not the whole predicted protein, but fragments from the highly-conserved regions of the selected genes. The fragments would also reduce expression and solubility problems associated with full length recombinant proteins. The resulting PCR products were run on 2% agarose gel and purified with Qiaquick gel extraction kit (cat no. 28704; Qiagen) according to the manufacturers’ protocol. The gel-extracted products were cloned in pJET1.2 vector (cat no. K1231; Thermo Fisher Scientific) using the CloneJET PCR cloning kit’s protocol.

Table S1

### 2.3 Expression and purification of recombinant proteins

The gene fragments were digested with *Bam*HI (site designed in the forward primer) and *Not*I from pJET1.2 vector and ligated in pET28a expression vector, which was used to transform BL21 (DE3) star and/or JM109 (DE3) *E. coli* strains. An overnight culture was generated by inoculating 50 ml of 2x YT medium (tryptone 16 g/liter; yeast extract, 10 g/liter; NaCl, 5.0 g/liter) containing 50 μg/ml kanamycin monosulphate (kanamycin A), with a loop of *E. coli* cells containing pET-28a with the cloned *T. parva* gene fragments and incubated at 37 °C with shaking. The next morning, 5 ml of this overnight culture was added to 500 ml of 2x YT containing 50 μg/ml kanamycin and incubated at 37 °C with shaking until the cells reached A600 between 0.5 and 0.7 then isopropyl-1-thio-b-d-galactopyranoside (IPTG) added to a final concentration of 2 mM. Samples of 2 ml were taken just before induction (non-induced control), 4 h post induction and overnight post induction and were used to screen expression levels. Cells were harvested by centrifugation and then sonicated in buffer B (100 mM NaH_2_PO_4_, 10 mM Tris·Cl, 8 M urea, pH 8.0). The resulting supernatant was bound to Ni-sepharose (GE Healthcare Life Sciences, cat no. 17-5318-01) overnight and column purified. The column was washed with buffer C (buffer B at pH 6.3) and protein eluted in elution buffer (0.1 M NaH_2_PO_4_, 0.3 M NaCl, 10% glycerol, pH 3) and dialyzed extensively in PBS. Two proteins (TpMuguga_01g00972 and TpMuguga_01g00095) could not be eluted successfully from the resin. These resin bound proteins were denatured, run on SDS-PAGE gels and stained with Nile Red (8 μg/ml final concentration in deionized water) as described in (Daban et al., 1996). The proteins were visualized by UV trans-illuminator and cut from the gel. The gel pieces with the proteins were ground using mortar and pestle, dissolved in PBS, vortexed briefly then centrifuged and the supernatants containing the were proteins retained.

### 2.4 Generation of murine antibodies to purified recombinant protein

All animal procedures described in this article were approved by ILRI’s Institute Animal Care and Use Committee (IACUC File Number 2015.16). The mice used for production of polyclonal antibodies were Swiss mice, 6–8 weeks old and each recombinant antigen was used for immunization of two mice. Blood was collected from the tails of the mice (pre-immunization control) and each mouse inoculated intraperitoneally with 75 μg recombinant protein (in PBS) and Freund’s adjuvant, Incomplete (cat no. F5506) mixture. Antigen and adjuvant were mixed on equal volume basis. Boosting was performed bi-weekly until day 42. Blood was collected by cardiac puncture after cervical dislocation at the end of the experiment.

### 2.5 Assessment of immune responses via ELISA

ELISA was performed by coating the Nunc Maxisorp 96 well plates (cat no.439454) with 100 μl/well recombinant protein (500 ng/ml) in PBS and incubated overnight at 4 °C. The coating solution was flicked out and the inverted plates slapped onto paper towels and washed with 150 μl of PBS-T20 (0.1% tween 20 in PBS) four times. Blocking was then done by adding 150 μl of blocking buffer (0.2% casein in PBS-T20) per well and incubated for 1 h. at 37 °C. The blocking buffer was flicked out and washed as before, four times with 150 μl of PBS-T20. Corresponding sera diluted from 1/33 to 1/72900 were added to the wells at 100 μl per well and incubated for 1.5 h at 37 °C. The sera were flicked out and washed four times as before with 150 μl of PBS-T20. Secondary antibody, anti-mouse IgG peroxidase produced in rabbit (Sigma A9044), was added at 1/1000 dilution, 100 μl/well. The reactions were revealed using the substrate 2, 2′-azino-di-[3-ethyl-benzothiazoline-6 sulfonic acid] diammonium salt (ABTS). Optical density was read at 405 nm on a microplate reader (Labsystems Multiskan MCC 340, Helsinki, Finland).

### 2.6 Sporozoite neutralization assays

The neutralization of sporozoite infectivity was performed using a slight modification of a previously described method (Musoke et al., 1992). The procedure for sporozoites production and infection rates assessment has been described before (Patel et al., 2016). In each well of a 96-well microtiter plate, 5 × 10^5^ bovine peripheral blood mononuclear cells isolated from uninfected bovine blood by Ficoll-Paque density gradient centrifugation were added and incubated for 2hr at 37 °C, 5% C02. A sporozoite suspension obtained from 1050 infected acini (approximately 3.7 × 10^7^ sporozoites, mean infection rate of 28,570 sporozoites/acinus) in 100 μl RPMI 1640 medium with 7.5% fetal bovine serum and 5% DMSO was diluted 100 times and 100 μl with approximately 3.7 × 10^5^ sporozoites/well added to various dilutions (1/10, 1/100 and 1/1000) of pre- and day 56 post-immunization sera (poled for each antigen, heat inactivated at 56 °C for 30 min) and positive control monoclonal antibody (ARIV21.4) with neat concentration of 670 μg/ml, previously generated against the major sporozoite protein p67 and incubated for 10 min at 37 °C, 5% C0_2_. Giemsa-stained cytospin smears prepared from each well were examined for the presence of schizonts at day 14. One hundred cells from each well were counted and the percentage of cells containing schizonts was determined. Percent reduction in infection intensity were calculated relative to the control (PBMCs incubated with sporozoites only). The assays were performed in triplicate and scored by a blinded operator. All analyses were performed using GraphPad PRISM^®^ version 7.01 with alpha = 0.05. For each antiserum, the Mann–Whitney *U* test was used to assess the differences in neutralizing ability of the recombinant antisera in relation to positive control monoclonal antibody, antip67c.

## 3 Results

### 3.1 Selection and *in silico* analysis of proteins

Sequence analysis of the predicted *T. parva* proteome of 4085 genes with PredGPI (Pierleoni et al., 2008) revealed 21 highly probable GPI anchored proteins. The top 10 highly probable proteins were selected for this study. Analysis of these 10 proteins with SignalP 4.1 (Petersen et al., 2011) revealed signal peptides for all the proteins except one, TpMuguga_04g02375 (Table 1).

**Table 1:** The 10-selected putative *T. parva* surface proteins.

ORF locus tag (antisera)	Annotation	cDNA amplicon size (bp)	Expressed protein size (kDa)**	Full protein size (kDa)	Identified by LC–MS/MS
TpMuguga_04g00437 (anti437)	104 kDa antigen (p104)	312	12	104	Yes
TpMuguga_01g00939 (anti939)	hypothetical protein (gp34)	375	15	34	Yes
TpMuguga_01g00876 (anti876)	hypothetical protein	274	10	13	Yes
TpMuguga_01g00095 (anti095)	hypothetical protein	331	12	28.6	No
TpMuguga_01g00575 (anti575)	hypothetical protein	366	14	197.4	No
TpMuguga_01g00972 (anti972)	hypothetical protein	320	12	37.8	Yes
TpMuguga_03g00844	hypothetical protein	330	NE	13.3	Yes
TpMuguga_02g00792	hypothetical protein	331	NE	15	Yes
TpMuguga_03g00136	hypothetical protein	310	NE	20.6	No
TpMuguga_04g02375*	hypothetical protein	296	NE	40	No

Selected *T. parva* proteins predicted to contain a C-terminal GPI anchor signal and/or an N-terminal signal peptide. Data presented include ORF locus tag with the corresponding antisera in brackets, annotation, cloned gene fragment size and corresponding expressed protein size, the full protein size and whether the protein was identified by mass spectrometry in the sporozoite proteome (Nyagwange et al., 2018). Hypothetical protein is of unknown function(s). GPI anchor predicted using the PredGPI (http://gpcr2.biocomp.unibo.it/predgpi/) and signal peptides predicted using the SignalP 4.1 server (http://www.cbs.dtu.dk/services/SignalP/).

(*) Reannotated from TP04_0030 and protein does not contain a predicted signal peptide; (**) Size excludes His-tag; (NE) Protein not expressed.

### 3.2 Cloning, expression and purification of recombinant proteins

Amplification of fragments of the selected genes by RT-PCR yielded amplicons of expected sizes ranging from 274 to 375 base pairs (Supplementary Fig. S1A). Fragments and not the whole proteins, were selected from the more conserved regions of the genes (Table 2). The conserved fragments would ensure broader protection but also avoid expression and solubility problems associated with the recombinant full-length proteins. All the fragments were inserted into pJET1.2 blunt vector and subsequently transferred to the expression plasmid pET28a (Supplementary Fig. S1B). All the expressed fragments were in pET28a except TpMuguga_01g00972, which was expressed in pGS-21a as GST fusion protein because expression with the pET28a vector was not successful (Supplementary Fig. S1C). All the recombinant proteins were expressed with a hexa-histidine tag that enables affinity purification by immobilized metal affinity chromatography (IMAC). Sequence analysis of the cloned gene fragments demonstrated 100% sequence identity with the published gene sequences (results not shown).

Fig. S1

**Table 2:** Nucleotide sequence polymorphism of open reading frames and recombinantly expressed fragments, in cattle and buffalo derived *T. parva* isolates. SNPs expressed as percentage of DNA sequence length for the whole gene (WG) sequence and the recombinantly expressed fragment (EF).

Isolate	TpMuguga_01g00095		TpMuguga_ 01g00575		TpMuguga_ 01g00876		TpMuguga_ 01g00939		TpMuguga_01g00972		TpMuguga_ 04g00437	
	WG	EF	WG	EF	WG	EF	WG	EF	WG	EF	WG	EF
ChitongoZ2	0	0	5.7	0.3	0	0	2.3	0.3	0.5	0	1.5	0
Entebbe	0	0	4.1	0.3	0	0	2.1	0.3	0.4	0	0.7	0
KateteB2	0	0	5.5	0.3	0.8	0	2.3	0.3	0.5	0	1.1	0
Katumba	0	0	6.0	1.9	2.6	0.4	2.6	1.6	0.5	0	0.8	0
Kiambu5	0	0	23.5	2.5	7.1	1.1	3.0	1.6	0.5	0	1.8	0
KiambuZ464/C12	0	0	7.6	2.5	2.6	1.1	2.7	1.6	0.5	0	1.5	0
MandaliZ22	0	0	4.4	0.3	0	0	2.1	0.3	0.4	0	0.6	0
Marikebuni	0	0	3.8	0.3	5.8	1.1	0.1	0.3	0.1	0	1.7	0
MugMar	0	0	3.7	0.3	6.1	1.1	0.1	0.3	0.1	0	1.7	0
MugUg	0	0	5.9	0.3	0.3	0	2.4	0.3	0.6	0	1.7	0
Muguga2*	0	0	0	0	0	0	0	0	0	0	0	0
Nyakizu	0.1	0	0.7	0.3	5.0	1.1	2.9	1.6	0.5	0	1.2	0
Serengeti	0	0	0	0	0	0	0	0	0	0	0	0
Uganda	0	0	5.9	0.3	0.3	0	2.4	0.3	0.6	0	1.7	0
Buffalo LAWR**	2.0	1.2	6.1	0.3	3.2	0.4	2.0	1.1	1.4	1.9	2.8	0.6
Buffalo Z5E5**	2.1	0.9	7.7	0.8	1.9	0.7	2.0	0.8	1.0	1.6	3.0	0.6

The isolates shown include cattle derived and two buffalo derived isolates (**) and a clone of the reference genome isolate (*).

**Fig. 1:**
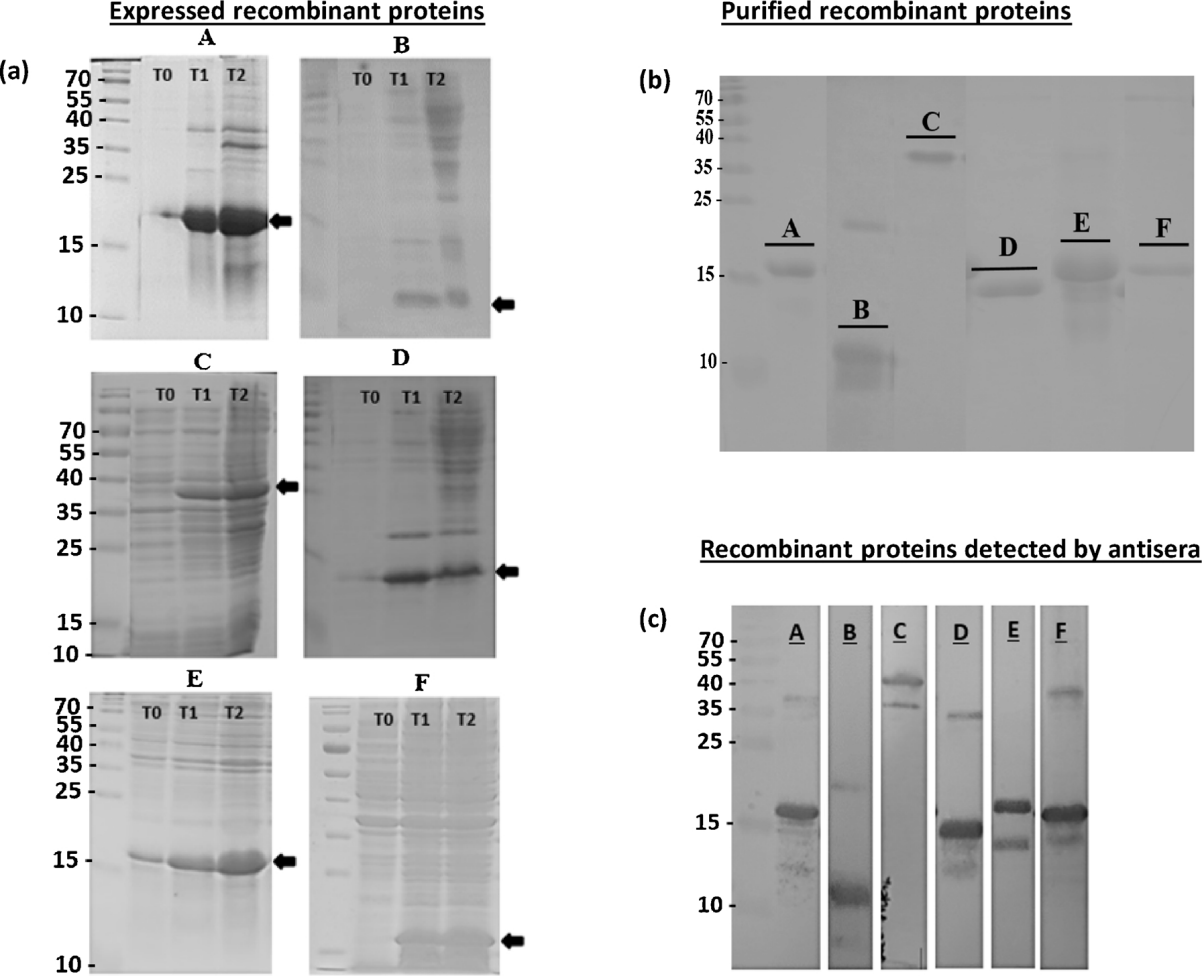
SDS-PAGE and western blot showing expressed (a), purified (b) and antisera detected (c) recombinant protein fragments. (a) bacteria lysate before IPTG induction (T0), 4 h (T1) and overnight (T2) post IPTG induction. (b) IMAC purified or gel extracted recombinant proteins are shown. Recombinant proteins include; TpMuguga_01g00939 (A), TpMuguga_01g00876 (B), TpMuguga_01g00972 (C), TpMuguga_01g00095 (D), TpMuguga_01g00575 (E) and TpMuguga_04g00437 (F).

Analysis by SDS-PAGE demonstrated that *E. coli* cells transformed with pET28a-inserts and pGS-21a – TpMuguga_01g00972 insert, expressed, considerable amounts of the six recombinant proteins after IPTG induction (Fig. 1a). Four constructs failed to express detectable recombinant protein (data not shown). All the expressed proteins were in the insoluble fractions (inclusion bodies) after cell lysis and were dissolved in 8 M urea buffer and purified on Ni^2+^ chelating sepharose beads, employing the 6xHis-tags. Four of the expressed proteins bound to the column and were successfully eluted by addition of elution buffer. Once eluted, the recombinant proteins remained soluble after removal of urea arising from the wash buffers by step dialysis against PBS at 4 °C (Fig. 1b). Two proteins, C and D could not be eluted successfully and were extracted from SDS-PAGE gels following Nile red staining (see Materials and methods) and remained soluble in PBS.

### 3.3 Analysis of antibodies to recombinant proteins

**Fig. 2:**
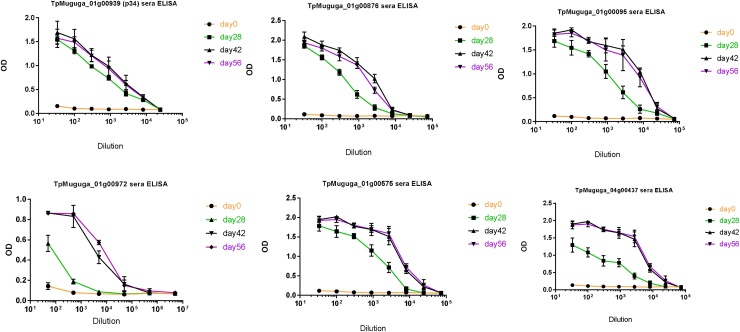
ELISA for antibody titres to the recombinant proteins. Immunization of mice was done at day 0 and three boosts performed biweekly thereafter. OD at 405 nm is shown relative to three-fold dilutions of the sera.

**Fig. 3:**
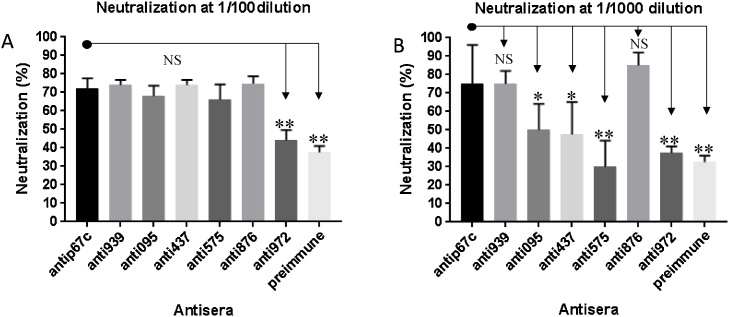
Summary of the *in vitro* neutralization of sporozoite infectivity. Monoclonal antibodies against p67c (positive control), normal mouse serum (pre-immune) and antisera against the six recombinant protein fragments were used for neutralization of sporozoites *in vitro*. Results are shown for sera − gene related to; TpMuguga_01g00939 (anti939), TpMuguga_01g00095 (anti095), TpMuguga_04g00437 (anti437), TpMuguga_01g00575 (anti575), TpMuguga_01g00876 (anti876) and TpMuguga_01g00972 (anti972). Antisera were diluted 1:100 (A) and 1:1000 (B). Antisera with significant differences to antip67c shown as * (p = 0.0152, anti095 and p = 0.0260, anti437), ** p = 0.0022, NS– not significant.

Each purified recombinant protein was used to immunize two mice. Sera from these mice showed indirect ELISA titres higher than 1000 (Fig. 2) and binding in immunoblots (Fig. 1c) to the corresponding recombinant proteins used for the immunizations. More importantly, when used in a 1:100 dilution all the antisera, apart from antisera 972 (Mann–Whitney *U* test p = 0.0022; Fig. 3A), were able to neutralize sporozoite infectivity to a similar extent as the positive control, a monoclonal antibody (ARIV21.4) previously generated against the major sporozoite protein p67. At a higher antiserum dilution of 1:1000 two antisera (anti095, p = 0.0152 and anti 437, p = 0.0260) still showed some neutralizing activity, although significantly lower than the antip67C. However, antisera 876 and 939 displayed similar or even higher sporozoite neutralizing activity than the anti-p67C positive control (Fig. 3B).

### 3.4 Conservation of selected gene fragments

Ideal vaccine candidate antigens should be conserved amongst the various isolates of *T. parva* to ensure broad protection following vaccination. We used the re-annotated *T. parva* genome sequence information (cited in Tretina et al., 2016) and identified single nucleotide polymorphisms (SNPs) in the six expressed genes for the whole gene (WG) sequences and the expressed fragments (EF). We found that most of the genes were conserved amongst the cattle derived isolates compared to the two buffalo derived isolates, Buffalo LAWR and Buffalo Z5E5, and that the EF are more conserved than the WG sequences (Table 2). For all the WG sequences except TpMuguga_01g00575 and TpMuguga_04g00437, non-synonymous SNPs made up <50% of the SNPs observed (data not shown). TpMuguga_01g00095 WG sequence was the most conserved antigen being totally conserved amongst the cattle-derived isolates with just a single synonymous SNP in Nyakizu isolate representing a SNP rate of 0.1% per WG sequence length. It was fairly conserved in the two-wild buffalo derived isolates − Buffalo LAWR (2%) and Buffalo Z5E5 (2.1%). TpMuguga_01g00575 WG sequence was the least conserved antigen having many SNPs per WG sequence relative to cattle derived isolates, Kiambu5 (23.5%) and KiambuZ464/C12 (7.6%) and also relative to the two buffalo derived isolates Buffalo LAWR (6.1%) and Buffalo Z5E5 (7.7%) (Table 2). Although a majority of the EF were conserved, polymorphism was observed amongst many of the WG sequences selected.

## 4 Discussion

GPI anchors are common attachment signals for surface proteins of parasites such as *Plasmodium, Trypanosomes*, *Toxoplasma,* etc. and many are promising vaccine candidate antigens (Ferguson, 1999; Ferguson MAJ and Hart, 2009). One such example is the circumsporozoite (CS) protein of *Plasmodium* which is the antigenic target of the malaria vaccine RTS, S (Lancet, 2015). Researchers have employed the strategy of targeting GPI anchored proteins for evaluation as candidate vaccine antigens. In this study, we also employed this strategy to select *T. parva* proteins predicted to contain GPI anchor signals for evaluation as vaccine candidates.

We selected a list of 10 genes with high probability of containing a GPI-anchored tail (Table 1). Using specific primers for the more conserved regions of the selected genes, we were able to synthesize cDNA of expected sizes in a one-step RTPCR reaction (Supplementary Fig. S1A). We have expressed six out of the 10 selected genes as recombinant proteins in *E. coli*. Five of the proteins were expressed with a His tag, which increases the molecular weight by approximately 1 kDa (Fig. 1). TpMuguga_01g00972 was expressed as a GST fusion protein, adding 26 kDa to the size resulting in a total size of 39 kDa (Fig. 1C). Although sequencing data showed that the cloned sequences were in-frame, we were not successful in expressing four of the 10 selected gene fragments even after transfer to various expression vectors (pET28a, pQE30 and pGS-21a) and *E. coli* strains, BL21(DE3) star and JM109(DE3).

The six expressed recombinant proteins were used to raise antisera in mice. The antisera were found to bind to the respective protein product of the cloned gene fragments (Fig. 1c) and most importantly, antisera against two of the recombinant proteins, TpMuguga_01g00876 and TpMuguga_01g00939, highly (>60%) neutralize sporozoite infectivity at 1000-fold dilution. Antisera against two additional proteins, TpMuguga_01g00095 and TpMuguga_04g00437, moderately (>30%) neutralize sporozoite infection of bovine PBMCs *in vitro* (Fig. 3B).

In this study, we tested gene fragments. It is tempting to speculate that antisera to full length proteins of the four antigens would produce higher neutralizing activities because of the longer sequence with putative additional epitopes. Therefore, it is desirable to test the full length recombinant proteins for immunogenicity. However, expression of long proteins is usually accompanied by solubility and expression problems of the recombinant proteins. To overcome expression problems, other expression systems could be employed or several shorter fragments comprising the full protein can be combined and evaluated.

Among the protein fragments that produce high sporozoite neutralizing antibodies is TpMuguga_01g00939, a protein previously referred to as gp34, that undergoes GPI modification when expressed in mammalian cells (Xue et al., 2010). Although originally reported as a schizont stage specific antigen (Xue et al., 2010), we have recently identified gp34 protein in the sporozoite proteome (Nyagwange et al., 2018). We therefore conclude that the protein is expressed in both parasite life-cycle stages. In the schizont stage, gp34 seems to play a role in parasite-host interaction during host cell division (Xue et al., 2010). Immunization with TpMuguga_04g00437, also known as p104 a sporozoite microneme/rhoptry protein (Ebel et al., 1999), resulted in mouse antisera that moderately neutralized sporozoite infection of bovine PBMCs *in vitro*. The p104 protein was originally identified by sporozoite neutralizing bovine antisera C16 (Iams et al., 1990), but was never evaluated as a vaccine candidate antigen (Nene et al., 2016). The p104 protein is also expressed at the surface of the schizont and recent evidence suggests a role for this protein in interacting with the host-cell mitotic machinery (Huber et al., 2017). With the results presented here, it appears that gp34 and p104 also play a role in the lymphocyte invasion process, perhaps through additional interactions with host cell microtubules during invasion.

Neutralization of parasite infection of host cells is one of the most important features of an anti-sporozoite vaccine candidate antigen, and in this study, we have identified four vaccine candidates that are able to induce sporozoite neutralizing antibodies. Two of these proteins (p104 and gp34) were identified before and two are completely new, including TpMuguga_01g00876 which produced antibodies inducing the strongest sporozoite neutralizing activity. However, following previous observations in which rats immunized with recombinant polymorphic immuno-dominant molecule (PIM) make neutralizing antibodies while cattle immunized with the same do not (Toye et al., 1995; Toye et al., 1996), it is important to raise and test bovine antibodies against these antigens to formally confirm their role as candidate vaccine antigens.

## Author contributions

JN, ET, VN and RP took part in conception and design of the study, JN, SM and BN in acquisition of data, JN, SH, ET, LS and RP in analysis and interpretation of data. JN in drafting the article, ET, LS and VN participated in revising it critically for important intellectual content. RP made final approval of the version to be submitted.

## Conflicts of interest

The authors declare no conflict of interest.
