# Supplementary material for: Antibodies to in silico selected GPI-anchored Theileria parva proteins neutralize sporozoite infection in vitro
Source: Vet Immunol Immunopathol. 2018 May;199:8–14. doi: 10.1016/j.vetimm.2018.03.004 (PMC5956992; doi:10.1016/j.vetimm.2018.03.004)
Supplement: Table S1 [file mmc2.docx]

**Supplementary Table S1a. Ten selected highly probable GPI-anchored T. parva proteins.** The six proteins expressed in *E. coli* are in yellow color.

| # | **Gene locus** | **Re-annotation** | **Protein product** | **GenBank Acc. No.** | **Signal P** |
| --- | --- | --- | --- | --- | --- |
| 1 | TP01_0095 | TpMuguga_01g00095 | hypothetical protein | XP_765622 | YES |
| 2 | TP01_0876 | TpMuguga_01g00876 | hypothetical protein | XP_766397 | YES |
| 3 | TP04_0437 | TpMuguga_04g00437 | 104 kDa antigen | XP_764072 | YES |
| 4 | TP01_0575 | TpMuguga_01g00575 | hypothetical protein | XP_766094 | YES |
| 5 | TP01_0972 | TpMuguga_01g00972 | hypothetical protein | XP_766493 | YES |
| 6 | TP01_0939 | TpMuguga_01g00939 | hypothetical protein, p34 | XP_766460 | YES |
| 7 | TP03_0844 | TpMuguga_03g00844 | hypothetical protein | XP_762968 | YES |
| 8 | TP02_0792 | TpMuguga_02g00792 | hypothetical protein | XP_765359 | YES |
| 9 | TP03_0136 | TpMuguga_03g00136 | hypothetical protein | XP_763154 | YES |
| 10 | TP04_0030 | TpMuguga_04g02375 | hypothetical protein | XP_763665 | NO |

**Supplementary Table S1b. Primers and sequences of selected genes.** Data shown include the ORF Locus tag, the forward (sense) and reverse (antisense) primers. The full length sequence of the genes is shown with fragments cloned for expression highlighted in yellow.

| **ORF Locus tag** | | **TpMuguga_01g00095** | |
| --- | --- | --- | --- |
| Primers | | GGGATCCGCCAAGGTAGATGCTGACGAATA (Sense) | |
|  |  | GCTGCAGCTAAAGGGACGCTGTTAAGAATGAA (Antisense) | |
| Sequence | | ATGAAGTCTTTAATTTTTGGTTTTTTGTTTTTAAAATTATCATTATGCTCAACATCAGGGTTTGAACTTGACGTTTCCACAAAATACCATGACCCAAACG | |
|  |  | CCGTTGTTACCACAGAGCTCAAATTAGAGGCAGAAACCAGAGCTGGACCATCCGTCAAGACAGTTGTGTTGTATGCTTCCATGTACGACGATGGTTTTGA | |
|  |  | CAAGGTTAAAGATGGCCAAAAGCCAATCTGGGAACCCGAGACTGGCTTCAAATCCGACTACGTCAAAGTATTTTATAAAGGTCTTGCTCCAGAGGCACTA | |
|  |  | \| AAGGTTGGACTTTTAGACAAAAACACTCTCACTTACAATGCCGAAGACCACTACGCCAGGTTCTACTTGAAAAAAGACCAAGACTGGG \| CCAAGGTAGATG \| \| --- \| --- \| | |
|  |  | \| CTGACGAATA \| CTTTGAATTTTTGGAGAAGAACGAGTACAAAAACCTGGTCCACAAAGAATTTGAAAAGGACGAAAAGTTCGAAAAACCTGACGACGAATG \| \| --- \| --- \| | |
|  |  | TATAATACAAGTCGGTAGGAAGTACACTCCAGACGAGGTACAGGCCTTCAAGAAATTATCAGACTCTAAAGGGCTCGTTAAAGTCGATCCTTTAGTCAAA | |
|  |  | \| CCCTCAGAATCTGAGGATACAAACTTGAGAAGAAGGCAAGGAGGCTCGGATTCATCGCCTGCTCCAACAGAGCCAGGTTCTCCTGACAAAAAGGGAAG \| TT \| \| --- \| --- \| | |
|  |  | \| CATTCTTAACAGCGTCCCT \| TTTACTCCCAATCGTCCTATTGTCATGTACTTTATTTAATTAA \| \| --- \| --- \| | |
| **ORF Locus tag** | | **TpMuguga_01g00876** | |
| Primers | | GGGATCCCGGTTCGGTTCCGATGATACA (Sense) | |
|  | | GCTGCAGCTAAAGTCTCCTGAACAGGATAAACAA (Antisense) | |
| Sequence | | \| ATATTAATTTATAAATGTGTACTATTTTTAAACATGTCGTATTATAAATCAAATAAACATCTATCGCAATCTACGGTATCGGATATTAACC \| GGTTCGGTT \| \| --- \| --- \| | |
|  | | \| CCGATGATACA \| TTCGTAGTTGACCTGGAATGCAAAGTTGATAGCTTAAAAAATATTAGTACGAATATGCACAATGAACTCAAAAAATCCAAGGCTCACTT \| \| --- \| --- \| | |
|  | | ATCTTCTGTGTCAAAAGTGTTCAATTCTGCTTCCCTATCGCTCAACACTACTCTGATAAATATGAATAAAATAGCCCGAGGATACGGGTTAAAACTCCCG | |
|  | | \| CTTTGGGCTCTTGCCTTAATTGTGGCATTTATTCTCCTTTTC \| TTGTTTATCCTGTTCAGGAGACT \| TTTAAGCAGATAA \| \| --- \| --- \| --- \| | |
| **ORF Locus tag** | | **TpMuguga_04g00437 (p104)** | |
| Primers | | CGGATCCGAGGTACTGGGTGATCCAAATC (Sense) | |
| T | | CCTGCAGCTAAAATCCCACCACAGAGGTAAC (Antisense) | |
| Sequence | | ATGAAGTTTCTTATTTTGCTATTTAACATTTTATGTTTGTTCCCAGTTTTGGCGGCAGACAACCACGGTGTTGGTCCTCAAGGGGCCTCCGGTGTAGATC | |
|  | | CTATAACTTTTGATATTAATTCAAATCAAACAGGCCCTGCATTTTTAACGGCCGTAGAGATGGCCGGTGTAAAATATCTTCAAGTACAACATGGCTCTAA | |
|  | | CGTAAATATCCATAGACTTGTTGAAGGGAACGTTGTAATTTGGGAGAATGCATCAACACCGTTGTATACCGGCGCAATCGTTACTAATAATGACGGGCCT | |
|  | | \| TATATGGCCTATGTT \| GAGGTACTGGGTGATCCAAATC \| TACAGTTTTTTATAAAATCAGGTGATGCTTGGGTGACCTTAAGTGAGCATGAATATTTAGCTA \| \| --- \| --- \| --- \| | |
|  | | AACTACAGGAAATCAGACAAGCAGTTCATATTGAATCAGTGTTTTCTCTTAACATGGCCTTCCAACTGGAAAATAACAAATATGAAGTTGAAACGCACGC | |
|  | | TAAAAATGGAGCAAATATGGTGACATTTATACCAAGGAATGGACATATTTGCAAAATGGTATATCATAAAAATGTCAGAATATACAAAGCAACGGGCAAT | |
|  | | \| GACACT \| GTTACCTCTGTGGTGGGATTT \| TTTAGGGGTTTGAGGTTATTGCTTATTAACGTTTTTAGTATTGACGATAATGGAATGATGAGTAACAGATACT \| \| --- \| --- \| --- \| | |
|  | | TTCAACATGTAGATGACAAATATGTTCCTATTAGTCAAAAAAACTATGAGACTGGTATAGTTAAATTAAAAGACTATAAGCATGCTTATCATCCTGTAGA | |
|  | | CTTGGATATTAAAGATATTGATTACACCATGTTCCATTTGGCTGATGCCACTTATCATGAACCTTGTTTCAAAATTATTCCAAACACAGGGTTTTGTATA | |
|  | | ACTAAGCTTTTTGACGGTGACCAAGTGCTCTATGAGAGTTTTAATCCCTTAATTCACTGTATCAATGAAGTACATATTTATGATAGAAATAACGGATCAA | |
|  | | TTATTTGTCTACATCTAAACTACAGCCCACCATCCTACAAAGCATACCTT | |
| **ORF Locus tag** | | **TpMuguga_01g00575** | |
| Primers | | GGGATCCCCACTAGGCCTGAATAGATATGG (Sense) | |
|  | | GCTGCAGCTAGGTGTTCCCGCTAGAGAAAT (Antisense) | |
| Sequence | | ATGATTTCAGCCAAAATTTATATATTCGGAGTGACATTTCTTATTTTGTCACTATTTACATTTAATTTTTTCATTCCAGGTGTCGGAGCTTGGAATGCTA | |
|  | | \| AAGATGTGCTT \| CCACTAGGCCTGAATAGATATGG \| TGAGGACCTTCACTTTTTCCATAAAGGGGGTGATTTGGACTCGTTCATCTCCTTGGAAAAGGTGCC \| \| --- \| --- \| --- \| | |
|  | | CGAATCCTCAGAGCTACGTTCCTCTTTGGCCCAGACAGCAGCTGATACCACTGCTCAGAGGACTGTAATCGCTGCTGGAACCAGCTCAGAAACCTGTACA | |
|  | | AGCTGGGAAGACGTCTCAGTTTGCTCCACTAAATCCACCATCAAATGCTTAGATAATGGGAAGCAACACTTGGAAATGTCTTGTTTCCTCATGAACACAG | |
|  | | \| TAACCTGGTCTGACTGGTCCCCCTGTGAAAACAATGTCCAGTACAGATTTGCACTTG \| ATTTCTCTAGCGGGAACACC \| CAGTATAGATACTGCGGTGATGG \| \| --- \| --- \| --- \| | |
|  | | AGCAGCTGCGCTGATGAATCGCATGAAGGGTCACACTCCCAAAAAGACGGAACAGGAGATCTTGAAGGAGCGTCTAGAGAAATACGTTAACCTAAACCGC | |
|  | | GAACTTCATAAAAAGAATGAAGAAGCGGAGAATCTTTACAATGCGTATATGCAAAAATATGCGAAGTTCTACGATCGGAATAAAGACCTAAATCGAAAAA | |
|  | | TTAAGGAATTTAATCAGAAAAATGATGAATTTGAAAAGCAAAAGCAGGCTCTAGACGACCAAAAATCCAAGTATGATTCTCTAAATTCTAATCTAGAGGT | |
|  | | ACGAACCAGAGATTTAGAGCGTGAAAAGGACAATTATAAGCGAAAAACTGATAAATTGGCCGAAATGGAACTTGAATTACAGGAAGATATTAAGTTTTAT | |
|  | | CACAAGAAACAGGATGAACTTAACGCTGAACGCTTGGCTTTACAAGACAAGGAAAAAGATTACGCTAAACGTTCCGAAGACTTGGAAAGTGAAAGGACCA | |
|  | | AAATTCTGGACAAAGATCGAAAATTAGAAGAGGAATTGGCCAGAATTAAA | |
| **ORF Locus tag** | | **TpMuguga_01g00972** | |
| Primers | | GGGATCCGGCTTCCACGCAAATAAAG (Sense) | |
|  | | GCTGCAGCTAGTATAGCAAGTGCTGGGATAG (Antisense) | |
| Sequence | | ATGTTTCCTTCTTTACTTTTTTATTTAAATCTTAGCTTTATTGGATTTTTCTGTAAATTAGTTGCAGGTGCCGATTTCACCCTCGATTTGGACCAAAAGA | |
|  | | \| GTTCAAATGATAAGTTACTAGTCAATCATACAACCCATTACGGTATAGCGACGATAGAATTCTATACCCAGGT \| CGGCTTCCACGCAAATAAAG \| TGGTCCA \| \| --- \| --- \| --- \| | |
|  | | GAGCACCTTGCCGGTCTGGGAAGCGGTCAATGACGAGCGTGTAGATGAGGCCGTGCTTTACTTTGGACGTGAAGCGCTTTCACTCTTATTTTTAAACGTG | |
|  | | AGACAAGCCGGGACTTCTATCTTCAAATACTATGTAAAAGTAAAGGGGAAATGGGCTGAAGTACTTGAGCCCGTAGCAAACTTCTTTGTCGACGTTGACA | |
|  | | \| GGTCATCATACACCGTAGAAGAGCTTGATTCCTTCGCCTTGGATATCATGTCAAAATATAACAGGGAAATG \| CTATCCCAGCACTTGCTATACC \| CACAATC \| \| --- \| --- \| --- \| | |
|  | | CTACAATTTCATTATCGAGCCTGTTGACTACTGTGCTCTCGAGATTACAAACCCTGCCAGATACTCAATTATGAAACAGACCATGATTGGTGCTGTGTTT | |
|  | | TCACATATTTATACCCCAAAGATTGACTTTGGGTTCAGTAAAGTATCTTATGACGGGCATGTTTTATGGTCTACTGATACGAATCGTGTTTTATGTTCCT | |
|  | | CAGTTTGGACTTATTTCAAGGGCGAATTTGATACCCTGATCCTTGTATCGTTATATGACTTTGTGTCCAAACAGGCTTCATTCTTACACTTCATGAAGAC | |
|  | | TGACTCTCTAATTGTTTCAACCTCTGAACCTGATTTCCAGAAACAAGTTGTTAAAATGACCTCCGCAGAATATGACGCCAAGGATATCAAACGTTATATC | |
|  | | AACGGTTACCTCACGGAACGCGATTTGGCTTTCGTCATATCCACCACTCTTCCCATCGTGGGCGTTCTCGCCACAGTACTATTTACATTCTAA | |
| **ORF Locus tag** | | **TpMuguga_01g00939 (gp34)** | |
| Primers | | CGGATCCTCGGCTCTGGAGGATAAGATTAC (Sense) | |
|  | | GCTGCAGCTACAGCTTCGTCAGGGATTTA (Antisense) | |
| Sequence | | \| ATGAAGTATATTTTATTTATTTTAATTTCAACTTGCGTGGTTTCCTCGGGGAAGT \| CGGCTCTGGAGGATAAGATTAC \| CAGATCCATGATACACACCGATT \| \| --- \| --- \| --- \| | |
|  | | ACGAAGTTAGAAGGTACATGACTAAGTGTTTTGAGGACTGTAAAAATTATATAAATGAGATTGATGATTATATTCAGAAAATTGAGGCCGCTAGGTTCAA | |
|  | | GGAAAGTTCCAAGAAACACGCCGCCAAATTCAGGCTCCACTATAAGGAATTCTATGGTGAATTCAAAAAATTAAGAACCACGTACGAAGCATATAAATTT | |
|  | | GATGAAAGCGATCCACTGTTTAGAAGTAAATTTCGCGAGAATCTCACTGTGTTTGATAAAACATTCAGGAAGGCCAGCTCCACATACTCTCGCTCCTTCA | |
|  | | \| GGAGCGTAAC \| TAAATCCCTGACGAAGCTGG \| ATGAAAGTAAAATGGCCTATATGGATACAGAAGTGTATAAAAAGTGTGAGAAGGCTACTTTAATAACAAC \| \| --- \| --- \| --- \| | |
|  | | TCACAGACTCAGGGCTATTGAACAAATGGTTAAAATATTAGAAATCAGAGGAGATGAATGCGGTCCCTTATCTAACAGAGAACAGTTCCACAAGGACGCC | |
|  | | ACTGATAAAATAAATCGTTTACGTGATGAAAGTAAGAAGGCGATAGATGAGTTCCCGGTAACAGTAGTCAACGAGGAGCAATATAACAAGCTGGGCGAAG | |
|  | | TTGAGGAACGAGTAAAGACCTTGTTCGAAACTGGAATTAAAACCCTGGAAGAGTTTGACGAAAATCTTCGGAAAATGTATGAATCTAAAGGAGGAAAATA | |
|  | | CAACGATGATTTTAGAAAATTAATTAAATTCTTTGAAAGA | |
| **ORF Locus tag** | | **TpMuguga_03g00844** | |
| Primers | | GGGATCCACTCAGGACCCGTTACATGAATA (Sense) | |
|  | | GCTGCAGCTAGATGCAGAAGATGAAGACAAAG (Antisense) | |
| Sequence | | \| ATGTCAGAGA \| CTCAGGACCCGTTACATGAATA \| TAAACTACAGCAGAAAGAGAACTACGAAGAACGTAATCTCATGAAAGTTGGAACACATGTCCTTCAAG \| \| --- \| --- \| --- \| | |
|  | | ACGATGTTTACACCGCTCTAGATCCCAACGATAAGTTAACAGGATCTGTATACTCCATCTCAGACTACAATCAAAGGAGCACTGGGAATGTTTTTTCTGT | |
|  | | GTTTTTTTCATCATTAAAGGATAAAATGACACGTCTATCGGATTTTGTGGTTCCAAGTTACTCGTCACTTACACCTGCGGAAGCGGCTTCTTCCACGGCC | |
|  | | \| TTTTTCGGAGTTTTACT \| CTTTGTCTTCATCTTCTGCATCC \| TATCGCTCTTTTAA \| \| --- \| --- \| --- \| | |
| **ORF Locus tag** | | **TpMuguga_03g00136** | |
| Primers | | CGGATCCCCAAAGGACTCACAAGATACC (Sense) | |
|  | | CAAGCTTCTACTCCAAGCTCTCGATCTTTCC (Antisense) | |
|  | | ATGAAATACACTCCAGTTGTTTTAAGTTTATTAACCATGGGCTTAGTAAAAGCCGCAGGTAAAGATAATTTGAAAGTCGTAAAGTCTGACGGCGAATACC | |
|  | | \| TCGAATGGAAAAAGGCTGCAGAGAAGGCAAAGACTGTTGGTGAAGCCAAGAAGACTGAATTTGATGAGCTTTTAGAAAAGAT \| ACCAAAGGACTCACAAGA \| \| --- \| --- \| | |
|  | | \| TACC \| CCCAAACTTGAGGTTACTAAGATCACCGAAGCCCTCAAAAAGGTCACTGATAAGGTAGCCGAAATCACTGCTCTTGAAGCAAAAGAATTCAAGGAA \| \| --- \| --- \| | |
|  | | GCCACTGATGAAAAGAAAAAGGAAGCATTAGGAGCCAAGGTCGGAAAAATGGGAGTTGAGTTGGCTGATCACATCATTGAATTAGACGGCATAAACAAGA | |
|  | | \| AGGCACATTCAGTTGCCTATAAGGAATTAGTAAAAGTCCACGGCCACGTCAAGATGCTTGAGTTCTTGAAG \| GGAAAGATCGAGAGCTTGGAG \| AAAGACGG \| \| --- \| --- \| --- \| | |
|  | | ATTTATGGCCGCCTCCCTTCTTTCAGCTTTGGCTCTTTCTGCTTTGGCTGTCGGTGCATCCTTCAATTAG | |
| **ORF Locus tag** | | **TpMuguga_02g00792** | |
| Primers | | CGGATCCTACGTTGGAGGACTTGGATTC (Sense) | |
|  | | GCTGCAGCTATGATGACAGTTGTGAGTCTGT (Antisense) | |
|  | | \| ATGGTAAATACGGTCGAGTTTGAGGAATTTGTCGCGAGAGCACTGGGCTTCACTAAGACCTCGGCGTTTAACCTTTCTTC \| ATACGTTGGAGGACTTGGAT \| \| --- \| --- \| | |
|  | | \| TC \| GGCACCGGGCTCTCCCTTGTCTTCACTAAAGCTCTCAGAACTTTCTTCGTACTCGCTTCCGGAACACTTCTCATCGTTCTCGTATTGAATAGGCATGG \| \| --- \| --- \| | |
|  | | ATATGTGAATGTGGATTTTAATAAGTTGGTGGGATATATAACAATTCGTATGTCGAGGTTAATGGAGTTGATATTGAGTAAGATGAACTTGGAGACTGGA | |
|  | | \| GATAACTTTGAATTTCTTAAAACGTTACATCAGTCAATG \| ACAGACTCACAACTGTCATCAG \| CAACATTCGGTCTTCTCACAGGGTTCACCTTAGGTCTTG \| \| --- \| --- \| --- \| | |
|  | | TACTTTTATAA | |
|  | |  | |
| **ORF Locus tag** | | **TpMuguga_04g02375** | |
| Primers | | GGGATCCATCAAATCCAATGTGGAAACAAAG (Sense) | |
|  | GCTGCAGCTAGTTAGTTGATGCACTAGGAAG (Antisense) | |  |
|  | | \| GAAGACCAACGAATCGTTGAATTCAAATCTGGAGATGTTTATCGAATTAATATAAT \| CAAATCCAATGTGGAAACAAAG \| TATATAGAGATAATTGTAACTA \| \| --- \| --- \| --- \| | |
|  | | GGAATGGAAATTTAAACATAACATACTTTGCAAAAAAACGAAATGAAACAACATGGAAAAAAATTACAAATTATGATTTTGACGATGCGTTGAGAAGTGA | |
|  | | AATTAATTACAAAGACAAATGTACTGAATGTAATGAAGTAGAAACAATAGTAGATGATCTACCATCAGAAATCAAGATCCAAATTAATCCGGAATTTAAA | |
|  | | \| ACCCAACCAATTGAAGATAATCCTAGAAAA \| CTTCCTAGTGCATCAACTAACG \| TGAATATAAAAGGTGGCGGATTACGAGTCAACTCTAGTTTCAAAATTA \| \| --- \| --- \| --- \| | |
|  | | ATAAAAGTTCCTTAGTGTTATTAGCAATTTTGTTAAGTCTAATTTAA | |
