# Supplementary material for: Antibodies to in silico selected GPI-anchored Theileria parva proteins neutralize sporozoite infection in vitro
Source: Vet Immunol Immunopathol. 2018 May;199:8–14. doi: 10.1016/j.vetimm.2018.03.004 (PMC5956992; doi:10.1016/j.vetimm.2018.03.004)
Supplement: Fig. S1 [file mmc1.pptx]

## Slide 1
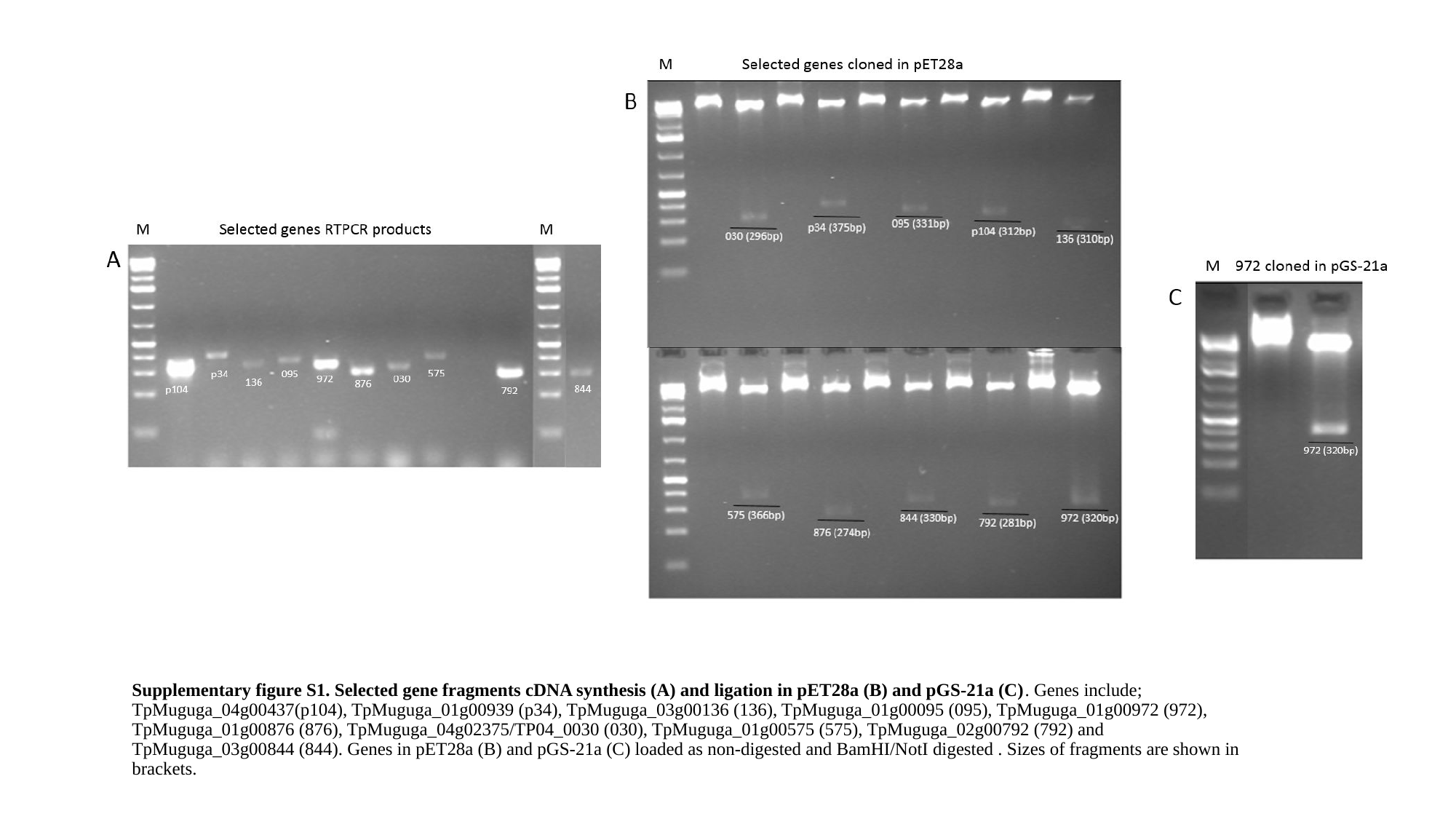

972 (320bp)
844 (330bp)
792 (281bp)
095 (331bp)
p104 (312bp)
030 (296bp)
Supplementary figure S1. Selected gene fragments cDNA synthesis (A) and ligation in pET28a (B) and pGS-21a (C). Genes include; TpMuguga_04g00437(p104), TpMuguga_01g00939 (p34), TpMuguga_03g00136 (136), TpMuguga_01g00095 (095), TpMuguga_01g00972 (972), TpMuguga_01g00876 (876), TpMuguga_04g02375/TP04_0030 (030), TpMuguga_01g00575 (575), TpMuguga_02g00792 (792) and TpMuguga_03g00844 (844). Genes in pET28a (B) and pGS-21a (C) loaded as non-digested and BamHI/NotI digested . Sizes of fragments are shown in brackets.
